# QoS-aware service composition based on context-free grammar and skyline in service function chaining using genetic algorithm

**DOI:** 10.7717/peerj-cs.603

**Published:** 2021-07-26

**Authors:** Pouya Khosravian, Sima Emadi, Ghasem Mirjalily, Behzad Zamani

**Affiliations:** 1 Department of Computer Engineering, Yazd Branch, Islamic Azad University, Yazd, Iran; 2 Department of Electrical Engineering, Yazd University, Yazd, Iran; 3 Department of Computer Engineering, Shahrekord Branch, Islamic Azad University, Shahrekord, Iran

**Keywords:** Service function chaining, Context-free grammar, Skyline method, Service composition, Genetic algorithm

## Abstract

Service function chaining (SFC) is a mechanism that allows service providers to combine various service functions and exploit the available virtual infrastructure. The best selection of virtual services in the network is essential for meeting user requirements and constraints. This paper proposes a novel approach to generate the optimal composition of the service functions. To this end, a genetic algorithm based on context-free grammar (CFG) that adheres to the Internet Engineering Task Force (IETF) standard and Skyline was developed to use in SFC. The IETF uses cases of the data center, security, and mobile network filtered out the invalid service chains, which resulted in reduced search space. The proposed genetic algorithm found the Skyline service chain instance with the highest quality. The genetic operations were defined to ensure that the service function chains generated in the algorithm process were standard. The experimental results showed that the proposed service composition method outperformed the other methods regarding the quality of service (QoS), running time, and time complexity metrics. Ultimately, the proposed CFG could be generalized to other SFC use cases.

## Introduction

Service function chaining is a composition of various service functions that must have crossed by network flows in a specific order (Yi et al., 2018). The commonplace method for such a service is a linear chain of at least one service function between two determined endpoints in the network. With appending service functions that divide network flows into different paths, the service construction can be made more complicated than an ordinary chain (Mirjalily & Lou, 2018). Since the copyright belongs to the designer, these services can be represented as directed graphs, including service functions (ETSI N, 2018). The service functions represent graph nodes, and the paths between the service functions represent graph links. These graphs are referred to as forwarding graphs according to the ETSI NFV (the Network Function Virtualization concept of the European Telecommunications Standards Institute).

In regard to resource allocation and network optimization plans, network operators require specific and compressed designs for the graphs that display the service structure and their procurement. After the joining process, the functions are defined and delivered correctly, and the regular graph presentation might be used to manifest the services (Bhamare et al., 2016). However, these presentations can rapidly become worthless when the particular process of joining the functions is not organized or is not appropriate for the functionality of the service (Rotsos et al., 2017). For instance, since there are no direct relations between the two functions used for the flow, the network can benefit from a resilience service presentation that enables an operator to combine the essential services more effectively. Also, IETF scenarios can be used to reduce network service compositions and essential services more effectively. Khosravian et al. (2020) propose the IETF-based Finite Automaton (FA) model to limit the space of the optimal service chain composition problem by considering the practical scenarios.

Classification term is defined as locally instantiated matching of traffic flows against policy for subsequent application of the required network service functions. The policy may be customer, network, or service-specific. The Internet Engineering Task Force (IETF) defines SFC as an ordered or partially ordered set of service functions and the ordering constraints that must be applied to the packets, frames, or flows selected as a result of classification (Quinn & Nadeau, 2015). The success of the service function chaining process is due to this definition. Disregard of the partial or total order established between the various virtual network functions (VNFs) during the chaining process can lead to the service delivery not requested by the user or nonsense services. Also, if the system takes too long to chain the service functions, even though the time to map and schedule the chained service is well managed, the QoS constraints may not be satisfied. Therefore, reducing the number of service compositions can be effective in this regard.

In this study, The IETF-based context-free grammar as a descriptive model is defined to evaluate the correctness of the service function chain structure. The IETF-based CFG application reduces the number of compositions by removing the invalid SFCs (Linz, 2011), and the Skyline method removes service with low QoS. The skyline reduces the search space and only focuses on interesting service functions not dominated by any other service. Recently, Skyline has been recognized as a new and popular paradigm to find the most relevant services (Bhamare et al., 2016). It is a promising method that reduces user decisions by offering only the most exciting services, and as a result, simplifies the selection process. However, Skyline allows for returning incomparable and conflicting results, and the user often encounters some difficulties in selecting a good service with a better compromise between the criteria of interest.

In this phase, the Skyline method concentrates on service QoS alone without the direct relations between the two services. So, the GA is utilized to select the best service compositions, considered the QoS of all the possible services. The GA initializes the candidates using a random method that ensures the service compositions accept by employing CFG and maintains that correctness by employing restricted genetic operators throughout the evolutionary process.

In summary, the contributions of this paper are as follows:

- Defining a context-free grammar model based on the data center, mobile, and security use cases defined by the IETF SFC working group to check the service chain validity.
- Using a Skyline method to select the proper service instance.
- Defining a genetic algorithm to select the best service chain instance.
- Analysis of the QoS, running time, and time complexity of the proposed method and comparing it with other methods.

This paper includes the following sections. A brief review of related work and researches is explained in the related work section. The optimum SFC selection problem section describes the problem of finding optimum SFC and QoS parameters. The SFC context-free grammar is defined by the Skyline method in the proposed meta-heuristic algorithm section. The performance evaluation of the proposed method is presented in the evaluation section. Finally, the conclusions are shown in the conclusions section.

## Related Work

Quinn and Nadeau reviewed the rules of service functions (SFs), such as firewalls and load balancers, in SFC (Quinn & Nadeau, 2015). Their paper also showed SFC applications with group functionality and provided documentation for the architecture. Halpern et al. proposed an architecture document for the specification, creation, and maintenance of SFC in a network (Halpern & Pignataro, 2015). They also included the architectural concepts, principles, rules, and components used in the construction of compound services through SFCs deployment, emphasizing standardization in the IETF. Mehraghdam et al. formulized the virtualization of network functions by grammar. They processed the deployment request and built virtual network function graphs that can be mapped to the network. They also discussed the deployment of SFCs with a focus on standardization in the IETF (Mehraghdam, Keller & Karl, 2014). Mehraghdam and Karl have shown that distributed cloud services were usually characterized by the custom functions chain (Mehraghdam & Karl, 2015). They created complex structures between the paths by specific types of streams. Next, a grammar was presented to describe the functional structures based on the data modeling language, which could easily translate them into a specific configuration of SFs.

Khosravian et al. (2019) use regular expressions and finite automata to create a grammar-based use case in the IETF. They proposed a regular grammar that can be used to eliminate the invalid SFCs. Subsequently, the grammar evaluation was performed via the Cocke–Younger–Kasami algorithm, and the number of service chain compositions was significantly reduced (Khosravian et al., 2019). Khosravian et al. proposed a finite automaton model that limits the solution space of the service composition problem by considering the practical use cases. Since the chaining rules depend on the substrate physical platform, their model acts based on the data center, mobile, and security use cases introduced by IETF. The finite automaton matcher showed that the model was a suitable tool for validating the composed service chain correctness (Khosravian et al., 2020). Mehraghdam & Karl (2016) proposed an innovative solution to select a set of compounds from different services based on the required resources. Their evaluation results showed that the selected composition was an example of possible structures, which could be optimally located on the network. However, SFC requires the assessment of a chain's correctness and reducing the number of service compositions. Dräxler & Karl (2017) proposed a heuristic selection method, which gives a Pareto optimal set of the possible compositions of services, and the feasible combinations of various services with respect to several optimization purposes. They also introduced a heuristic algorithm for the placement of service function composition. The algorithm focuses on locating the service elements with the shortest path and enough space to accommodate the services.

Yu & Bouguettaya (2011) recommended using dominance association between the service providers to locate a set of the best possible services assigned as a Skyline service. They presented suitable algorithms to prepare the Skyline service with a decreased searching area rather than investigating all the possible service compositions. Wang et al. (2020) proposed a fast and safe service composition method to combine the physical network, cyberspace, and social network. In this method, the skyline element computation was performed to decrease the solution area. Then, the variation ratio was applied to refine the elements with greater QoS variation. Ultimately, based on the user end-to-end QoS requirements, the optimal elements were chosen by maximizing the compatibility function. Ouadah et al. (2019) offered the composite method to rank-order the Skyline services. Their method combined many approaches used in multi-criteria decision making. The Skyline method was applied to decrease the search space and highlight the attractive services that were not governed by any other services. Kumar & Purohit (2016) used a two-layer architecture for web service selection, prefiltering followed by skyline selection. The K-means clustering technique is used for grouping the web services with similar Quality of Service (QoS). To finding the best answer to the QoS-based service composition problem, some methods can be used, such as Integer linear programming (ILP) techniques, called an optimization algorithm (Liang et al., 2019; Lodi & Nagarajan, 2019; Wang et al., 2019). But some heuristic methods like a genetic algorithm (GA) can be used, known as an approximation algorithm (Dwiardhika & Tachibana, 2019).

The composition of services can be performed manually. However, most candidates would cause this to be a very time-consuming process, especially when it is necessary to select the most suitable option among many services that offer the same functionality. This fundamental problem remains open, and it has been the focus of a growing body of research that aims to propose suitable techniques for performing automat service selection and composition. All of the available methods do not offer a rule-based structure. Also, service compositions include many compositions, most of which have the wrong arrangement and need to be reduced.

## Optimum SFC Selection Problem

Assuming that *S* is a set of all SFs, *c*_*n*_ *= (s*_*1*_, *s*_*2*_, *…, s*_*n*_*)* and *s*_*i*_ ϵ*S* is a service chain length *n* when *s*_*i*_ represents service in the set *S* in the *i*^*th*^ service chain position. Various qualitative characteristics have also been defined for each service. Different initializations of the features lead to the creation of service instances. According to the IETF SFC working group, service chains can be defined by a mobile network (Napper et al., 2018), a data center network (Surendra et al., 2017), and a secure network (Wang et al., 2017), as shown in Eqs. (1) to (3), respectively.

(1) }{}C_{n,mobile}=(\{bng, cmts, pgw, tls\}, c_{n-1})

(2) \eqalignb{C_{n,datacenter}=(\{(e-fw),(e-fw, adc), (e-fw, adc, a-fw), (woc, e-fw, adc, a-fw), \cr(woc, e-fw, mon, adc, a-fw), (woc, e-fw, mon, s-fw, adc, mon, a-fw)\}, \crc_{n-6})}

(3) }{}C_{n,security}=(\{(fw, lb, ips),(fw, tls, avc)\},c_{n-2})

Where *C*_*n,mobile*_, *C*_*n,datacenter*,_ and *C*_*n,security*_ represent the sets of all service chains length *n* in the mobile, data center, and security networks, respectively. The CFG generates all service chain instances based on IETF use cases. The service function chaining, *SFC*_*n*,_ represents a set of service chains length *n* under the CFG:

(4) }{}SFC_{n} =\{C_{n,mobile} , C_{n,datacenter} , C_{n,security}\}

Different service chain's lengths *n* can be created by considering the service instances in the graph nodes. Different service compositions are made in the form of the chain }{}I_{i,{j_i}}^{s{p_i}} where }{}I_{i,{j_i}}^{s{p_i}} represents the service instance *s*_*i*_^th^ in the *j*_*i*_^*th*^ node of the network, provided by the service provider *sp*_*i*_ and *i = 1, 2,…, n*. Therefore, there are several service chain instances for the service chain request *c*_*n*_ where }{}SI_{{s_i}}^{s{p_i}} represents the set of service instances equivalent to the service *s*_*i*_^th^ provided by the *sp*_*i*_^*th*^ service provider, and }{}N_{{s_i}}^{s{p_i}} shows the number of *i*^*th*^ service instances for the *sp*_*i*_^*th*^ service provider in the network. Then, the total number of requests for the service instance equals \; \mathop \prod \nolimits_{i = 1}^n \mathop \sum \nolimits_{s{p_i} = 1}^{s{p_i}} N_{{s_i}}^{s{p_i}},\; and the total number of service instances is\; SI = \mathop {\rm U} \nolimits_{i = 1}^n SI_{{S_i}}^{s{p_i}}. The request for the *p*^*th*^ service instance is equivalent to the request for the service chain *c*_*s*_ and is determined as follows:

(5) }{}r_{i}^{p}=(I_{1,{j_1}}^{s{p_1}},\; I_{2,{j_2}}^{s{p_2}},\; \ldots .\; ,I_{n,{j_n}}^{s{p_n}}), j_{i}=1,2,\ldots,N_{{s_i}}^{s{p_i}}

The goal is also to find a service chain instance that has the highest *Goal*:

(6) }{}{}_\; ^\; r_i^* = \mathop {\max }\limits_{r_i^p} Goa{l^p}

*r*_i_* is the best service chain instance, and *Goal*^p^ shows the quality of service chain instance *p*. Three qualitative parameters, namely service provider similarity, cost, and capacity for each service instance, are considered to select an appropriate service instance. The service provider similarity is defined as the ratio of the service chain provider similarity to all the service providers in the network. The service provider similarity of the service chain instance request, *r*_*i*_^*p*^, is obtained from Eq. (7):

(7) }{}Similarit{y^p} = 1 - \displaystyle{{\left| {unic\left( {\left\{ {I_{1,{j_1}}^{s{p_1}},\; I_{2,{j_2}}^{s{p_2}},\; \ldots .\; ,I_{n,{j_n}}^{s{p_n}}\; } \right\}} \right)} \right|} \over {{N_{SP}}}}

Where *Similarity*^*p*^ is the similarity with the *p*^*th*^ service instance provider, }{}I_{i,{j_i}}^{s{p_i}} is the *s*_*j*_^*th*^ service instance in the node }{}{V_{{j_i}}} of the service provider *sp*_*i*_, *unic(a)* represents the set of unique elements *a*, and *N*_*SP*_ is the total number of service providers in the network. The capacity that a service chain needs to transfer data is the capacity of the service chain. The capacity of the service chain instance *r*_*i*_^*p*^ is obtained from Eq. (8):

(8) }{}Capacit{y^p} = \mathop \sum \limits_{i = 1}^n Capacity_{{I_{i,{j_i}}}}^{s{p_i}}

Where }{}Capacity_{{I_{i,{j_i}}}}^{s{p_i}} is the capacity of *s*_*j*_^*th*^ service instance in the node }{}{V_{{j_i}}}of the service provider *sp*_*i*_. The service chain is the cost required for assigning service to a service chain. The cost of the service chain instance, *r*_*i*_^*p*^, is obtained from Eq. (9):

(9) }{}Cos{t^p} = \mathop \sum \limits_{i = 1}^n Cost_{{I_{i,{j_i}}}}^{s{p_i}}

Where }{}{Cost}_{{I_{{\rm i},{{\rm j}_i}}}}^{s{p_i}} is the cost of the *s*_*i*_^*th*^ service instance in the node *V*_*ji*_ of the service provider *sp*_*i*_. *Goal*^p^ explains a size for the effectiveness of services in the service chain that is obtained from Eq. (10) for the service chain instance *r*_*i*_^*p*^:

(10) }{}Goa{l^p} = Normal\left( {\displaystyle{{Similarit{y^p}} \over {Cos{t^p} + Capacit{y^p}}}} \right)

All notations and their descriptions are introduced in Table 1.

**Table 1:** Notations and their descriptions.

Description	Notation
The set of all SFs	*S*
Length	*n*
Service chain length n	*C*_*n*_
S^th^ service provider	}{}S{P_S}
The set of }{}{V_{{j_i}}} nodes for the service provider *sp*_*i*_	}{}V_{sp}^S
The service instance *s*_*i*_^th^ in the *j*_*i*_^*th*^ node of the network, provided by the service provider *sp*_*i*_	}{}I_{i,{j_i}}^{s{p_i}}
The set of service instancezs equivalent to the service *s*_*i*_^th^ provided by the *sp*_*i*_^*th*^ service provider	}{}SI_{{s_i}}^{s{p_i}}
Number of *i*^*th*^ service instances for the *sp*_*i*_^*th*^ service provider	}{}N_{{s_i}}^{s{p_i}}
The total number of service providers	}{}N*sp*
The total number of service instances	}{}SI
The request for the *p*^*th*^ service instance is equivalent to the request for the service chain *c*_*s*_	*r*_*i*_^*p*^
The best service chain instance	*r*_i_^*^
The capacity of *s*_*j*_^*th*^ service instance in the node }{}{V_{{j_i}}}of the service provider *sp*_*i*_	}{}Capacity_{{I_{i,{j_i}}}}^{s{p_i}}
The cost of the *s*_*i*_^*th*^ service instance in the node *V*_*ji*_ of the service provider *sp*_*i*_	}{}Cost_{{I_{i,{j_i}}}}^{s{p_i}}
Explains a size for the effectiveness of services in the service chain	*Goal*^*p*^
Similarity with the *p*^*th*^ service instance provider and }{}I_{i,{j_i}}^{s{p_i}} is the *s*_*j*_^*th*^ service instance in the node }{}{V_{{j_i}}} of the service provider *sp*_*i*_	*Similarity*^*p*^
The proposed CFG	*CFG*
The set of service chains length *n* in accordance with the CFG	*SFC*_*n*_
The request for the *p*^*th*^ Skyline service instance is equivalent to the request for the service chain *c*_*s*_	*sky*_*i*_^*p*^
The Skyline service instance *s*_*i*_, in node j_i_ graphs given by the service provider *sp*_*i*_	}{}sky_{i,{j_i}}^{s{p_i}}
The tournament selection operator	*t*
The maximum generation	*maxGeneration*
The set of Skyline services	*Sky*
The initial population size	*P*_*g*_
The performing crossover operator probability	*P*_*c*_
The performing mutation operator probability	*P*_*m*_
Best chromosome in the set r^p^ based on the quality of service (*Goal*^*p*^)	*bestChromosome* *(r*^*p*^_,_ *Goal*^*p*^*)*
Generate different (random) chains by traversing various rules of the proposed CFG	*randomParser*
The random integer between *a*, and *b*	*random(a, b)*
The random decimal number between 0 and 1	*rand*
The set of unique elements *a*	*unic(a)*

## The proposed meta heuristic algorithm

### Skyline method

Skyline is a method that selects multiple standards based on an appropriate scoring function. Skyline consists of all the nearest Pareto points that do not dominate by other data points. Although the number of return points may be critical, it does not require any elevators to return to its nearest neighbors. In the worst-case scenario, all the data are restored. It is advisable to use more flexible and customizable extensions to meet the diverse needs of the users. Skyline, recognized for its algorithmic or Pareto geometry in business management, is essential for several purposes. It obtains Pareto’s optimum set, which indicates that these points cannot be controlled elsewhere in the dataset (Yu et al., 2019). Although the exact optimal point depends on specific criteria, Skyline can provide a set of candidates to remove unauthorized ones from the dataset when the optimal solution is consulted.

This section aims to select a set of services from among all services with the highest overall efficiency in providing all the particularized restrictions. In this regard, the service with the highest performance neglects to present a fit solution because it does not confirm that all the limitations are provided. Therefore, it is essential to find various compositions of services from any set. However, not all services are possible suitors for the answer. The primary aim of the proposed method is to make a Skyline query on the services of each group to detect the services that may be suitable for the composition. Next, it can narrow down the exploration area. Skyline queries are quickly defined to explain how they are utilized in the proposed method. It can be understood that one service dominates another service, namely *S*_*j*_. If *S*_*i*_ is appropriate or equivalent to *S*_*j*_ in all dimensions, then it is strictly suitable in the smallest dimension where *S* is a set of services; *S*_*i*_ and *S*_*j*_ are members of this set, *QoS* is a set of their quality parameters:

(11) \eqalignb{\forall k \;\; [1, \;|\; QoS \;|] \;:\; QoS\;_{k}(s_{i})\; < =  \;QoS\;_{k}(s_{j})\; {\rm and}\; \exists k \;\; [1, \;| \;QoS\; |] :QoS\;_{k}(s_{i})\; \lt\;QoS\;_{k}(s_{j})\cr

Therefore, a Skyline query selects the appropriate services for all the dimensions. Later, dominance connections are employed among the services based on their QoS properties. They are applied to recognize and cut services that are dominated by others. It can be noted that the Skyline services show many trade-offs among the QoS parameters; consequently, they are not homogeneous to the others because there is no pre-defined popular plan regarding the appropriate significance of these parameters. The use of Skyline services of any type needs pairwise relations of the QoS vectors of the emulated services. If there are many competing services, this process may be costly in terms of computing time. Various practical algorithms have been suggested to perform Skyline computation (Han, Wang & Lai, 2019; Liu et al., 2019). The process of providing Skyline services is independent of any particular service demands or practice contexts, and it does not need to be addressed online at demand time.

As a result, the possible techniques are applied to provide Skyline services offline until it stimulates the service selection method later at demand time. To this end, any service broker can handle the list of Skyline services of any type in its repository file. This file is refreshed every time a service registers, defects, or refreshes its QoS data in the repository. Whenever the service broker accepts a service request, the Skyline services of the adapted services are granted on demand. If the corresponding services are dispersed in a set of service brokers, the service request has a horizon that any broker can define. Then, the retrieved local Skyline services must be connected to compose a universal Skyline (Han, Wang & Lai, 2019; Liu et al., 2019).

An integer linear programming (ILP) technique can be applied to answer the QoS-based service composition problem, called the restriction optimization problem (Liang et al., 2019; Lodi & Nagarajan, 2019; Wang et al., 2019). Then, each ILP solver can be appropriated for this plan; however, several variables in this model belong to multiple service competitors, it can only be carried out efficiently for meager examples. To deal with this restriction removing all non-Skyline services from the ILP model to limit its search space to the shortest possible. By highlighting the only Skyline in all of the services, the selection method is accelerated when it can still formally detect the optimal selection. Let *SFC = {S*_*1*_, *. . . , S*_*n*_*}* be the optimal solution to an addressed demand, *i.e.*, the composing service that passes all the detailed limitations and enlarges the total utility. After that, all the client services of *SFC* will refer to the Skyline of a similar service where Si indicates the number of services.

(12) \forall s_{i} \;\in\; SFC:\; s_{i} \;\in\; SKY_{Si}

The Skyline services of any service are refined to improve the performance of the QoS-based service selection algorithms. Although the Skyline size can be different for any dataset, it entirely refers to the dispensing of the QoS data and the connections among the many QoS parameters. The main emerging competition will recognize a set of Skyline indicative services that adequately describes all the lags in the different QoS parameters. It will be possible to find a solution that overcomes the restrictions and includes a rate of efficiency. This competition chooses from the nominees. The number of nomination services must be large enough to find the answer to the request, and the number of nominee services must be small enough to provide acceptable calculations. Specific algorithms based on Skyline and CFG are suggested in the next section to meet this challenge.

### The proposed algorithm

**Figure 1:**
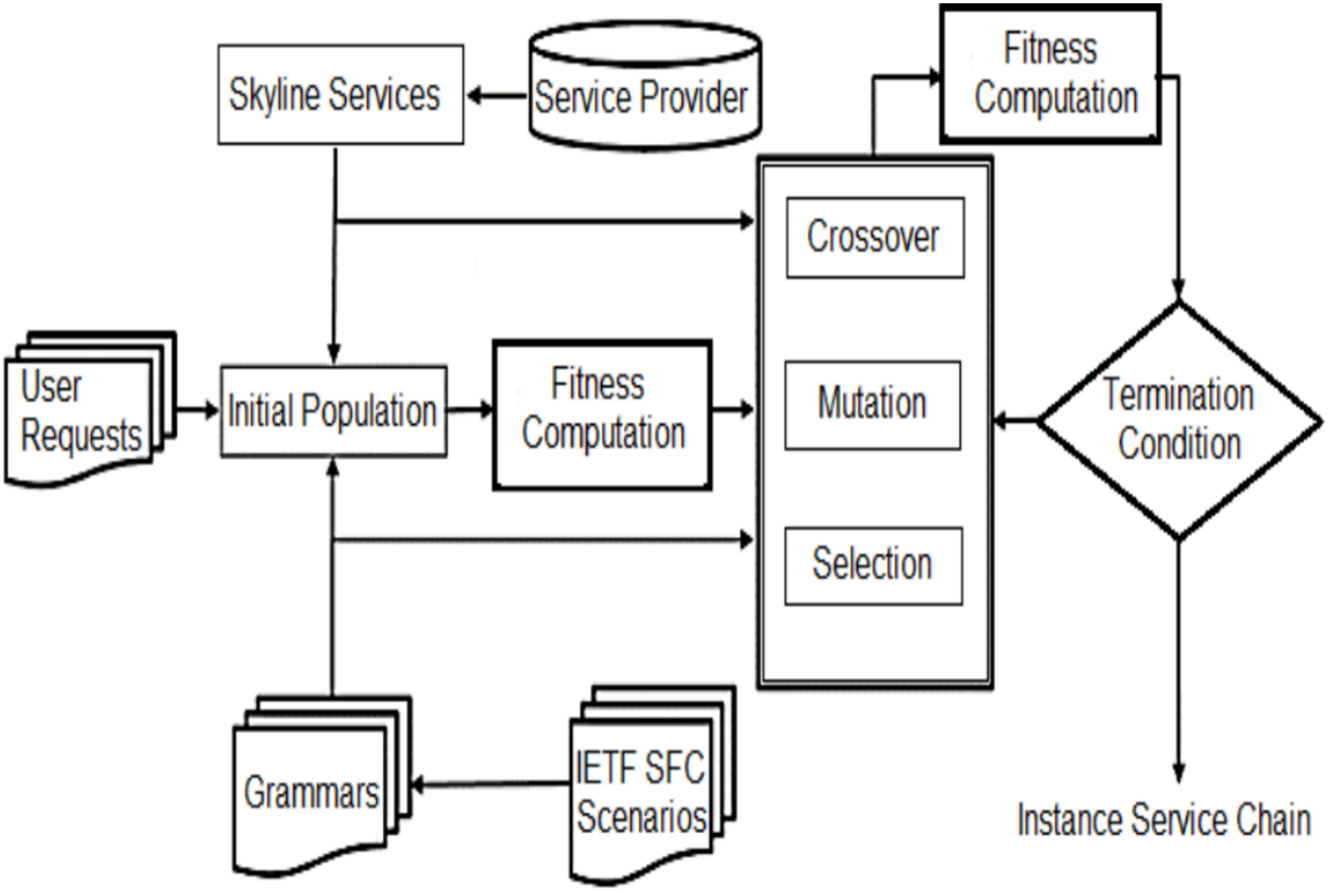
The proposed method block diagram.

The purpose of service composition is to select a set of services that can maximize productivity and meet all specified limitations. Note that choosing the highest value from each service class does not provide the right solution, as it does not prove that all constraints will be satisfied. Hence, various service compositions from each class should be maintained. Nonetheless, not all services may be possible candidates for the solution. Thus, instead of selecting all the service instances, only service instances through the Skyline technique are given to the algorithm. In this paper, inter-service dimensions are defined and exploited based on their QoS features, which are utilized to identify and prune services in one service class dominated by other services in the same class. Fig. 1 shows the block diagram of the proposed method.

Regarding the selection of service through the Skyline technique, *S*_*i*_ is better than *S*_*j*_ if all of the quality parameters of service *S*_*i*_ are better than those of service *S*_*j*_ and better in at least one parameter in *QoS* (Eq. (11)). The *QoS(s*_*i*_*)* is obtained from Eq. (13) as follows:

(13) \eqalignb{QoS \;(S_{i})\; =\; Normal([Availability(S_{i}) \;+\; Reliability(S_{i}) \;+\; Throughput(S_{i}) \;\cr+\; Reputation(S_{i}) \;+\; Bandwidth(S_{i})\; +\; Delivery(S_{i})]/[ Latency(S_{i}) \;\cr+\;ResponseTime(S_{i}) \;+\;Price(S_{i})])}

Where *Availability(S*_*i*_*)* is the availability of the service *S*_*i*_, *Reliability(S*_*i*_*)* is the reliability of the service *Si, Throughput(S*_*i*_*)* is the throughput of the service *Si*, *Reputation(S*_*i*_*)* is the reputation of the service *Si*, *ResponseTime(S*_*i*_*)* is the response time of the service *S*_*i*_, *Price(S*_*i*_*)* is the price of the service *S*_*i*_, *Bandwidth(S*_*i*_*)* is the bandwidth of the service *S*_*i*_, *Delivery(S*_*i*_*)* is the delivery of the service *S*_*i*_, and *Latency(S*_*i*_*)* is the latency of the service *S*_*i*_. The Skyline service request *p*, which is equivalent to the *C*_*n*_ service chain request, is determined by the following equation:

(14) \eqalignb{Sky_{S}\; = \; \{s_{i}\;\; S| \exists\;sj \;\; S:\; s_{j}\;<\; s_{i}\},\; sky_{i}^{p}\;\cr=\;(sky_{1,{j_1}}^{s{p_1}},\; sky_{2,{j_2}}^{s{p_2}},\; \ldots .\; ,sky_{n,{j_n}}^{s{p_n}}) , j_{i}\;=\;1,2,\ldots,N_{{s_i}}^{s{p_i}}}

Where }{}sky_{i,{j_i}}^{s{p_i}} means the Skyline service instance *s*_*i*_, in node j_i_ graphs given by the service provider *sp*_*i*_ when i = 1, 2, 3, …, n. The goal is to find a service chain instance that has the highest *Goal*:

(15) }{}{}_\; ^\; sky_i^* = \mathop {\max }\limits_{sky_i^p} Goa{l^p}

Furthermore, *sky*_i_* is the best Skyline service chain instance, and *Goal*^*p*^ is the quality of the Skyline service chain instance *p*.

Since this is an optimization problem, the GA can be employed to solve it. Also, the grammar can reduce the number of service compositions and search space by removing the invalid SFCs (Khosravian et al., 2019; Khosravian et al., 2020). Therefore, the GA is used along with the CFG to create service chain instances. The proposed method is shown as Algorithm 1, in which *S, n, CFG, P*_*m*_, *P*_*c*_, *P*_*g*_, *Sky, maxGeneration*, and *N*_*SP*_ are the input parameters, and sky* is the algorithm output. *N*_*sp*_ is the total number of service providers equal to \mathop \sum \nolimits_{i = 1}^n \mathop \sum \nolimits_{s{p_i} = 1}^{s{p_i}} N_{{s_i}}^{s{p_i}}, and *t* is the tournament selection operator. Also, *maxGeneration* is a maximum generation, and *Sky* is the set of Skyline services when *P*_*g*_ shows the initial population size. *P*_*m*_ is the performing mutation operator probability, and *P*_*c*_ is the performing crossover operator probability; *CFG* shows the proposed CFG when *n* is the service chain length, and *S* indicates the set of services.

**Algorithm 1:** Genetic-CFG-Service Chain composition.

*Input: S, n, CFG, P*_*m*_, *P*_*c*_, *P*_*g*_, *Sky, maxGeneration, N*_*SP*_, *t*
Output: sky*
*sky*^*p*^ *← initPopulation(CFG, n, P*_*g*_*)*
*itter ← 0*
*Goal*_*p*_ *← fitnessFunction(sky*_*p*_, *N*_*SP*_*)*
*while (itter < maxGeneration) do*
* sky*^*temp*^ *← crossover(sky*^*p*^, *P*_*c*_, *CFG, S, n)*
* sky*^*temp*^ *← mutation (sky*^*temp*^, *Pm, Sky, n)*
* Goal*^*temp*^ *← fitnessFunction(sky*^*temp*^, *N*_*sp*_*)*
* [sky*^*p*^, *Goal*^*p*^*] ← selection(sky*^*p*^, *Goal*^*p*^, *sky*^*temp*^, *Goal*^*temp*^, *t)*
* itter ← itter + 1*
*endwhile*
sky*← bestChromosome(sky^*p*^, *Goal*^*p*^*)*
return sky*

The function *bestChromosome(sky*^*p*^, *Goal*^*p*^*)* returns the best chromosome in the set *r*^*p*^ based on the quality of service (*Goal*^*p*^). The chromosome is an array length *n*, in which each entry represents a service instance. The *initPopulation* in Algorithm 2 generates the initial population. In all the steps of the genetic algorithm, the population contains chromosomes (Which each chromosome is equal to the service instance chain) accepted by the CFG. The function *randomParser* in this algorithm is employed to generate different (random) chains by traversing the various rules of the proposed CFG.

**Algorithm 2:** initPopulation.

*Input: CFG, n, P*_*g*_, *Sky*
*Output: sky*^*p*^
*sky*^*p*^ *←ø*
*for i = 1 to P*_*g*_
** }{}sk{y^p} \leftarrow sk{y^p} \cup randomParser\left( {CFG,\; n,\; Sky} \right)*endfor*
*return* }{}sk{y^p}

Algorithms 3 and 4 show crossover and mutation operators in genetic algorithms, respectively. In the proposed crossover operator, the one-point crossover is repeated until the CFG accepts the generated chromosomes. Repeat the crossover operator until the *P*_*g*_ new chromosomes are generated. The randomly selected index in the mutation operator is equivalent to the service instance. Then, this service instance is replaced by another service instance of the same service to ensure that the CFG still accepts the service chain instance and that there is no need to control the chain. The function *random (a, b)* in Algorithm 4 generates a random integer between *a* and *b*, and the variable *rand* is a random decimal number between 0 and 1.

**Algorithm 3:** Crossover.

*Input: sky*^*p*^, *P*_*c*_, *CFG, S, n*
*Output: sky*^*temp*^
*sky*^*temp*^ *←ø*
*for h = 1 to P*_*g*_*/2*
* i = random(1, P*_*g*_*)*
* while (i <> j) do*
* j = random(1, P*_*g*_*)*
* endwhile*
* if rand < P*_*c*_ *then*
* [*}{}sky_i^t, }{}sky_j^t*] ← onePointCrossover(*}{}sky_i^p, }{}sky_j^p*)*
* while ((not accept(CFG*, }{}sky_i^t*)or(not accept(CFG*, }{}sky_j^t*)) do*
* [*}{}sky_i^t, }{}sky_j^t*] ← onePointCrossover(*}{}sky_i^p, }{}sky_j^p*)*
* endwhile*
** \; \; sk{y^{temp}} \leftarrow sk{y^{temp}} \cup \left\{ {sky_i^t,\; sky_j^t} \right\} * else*
** \; sk{y^{temp}} \leftarrow sk{y^{temp}} \cup \left\{ {sky_i^{temp},\; sky_j^{temp}} \right\} * endif*
*endfor*
*return sky*^*temp*^

**Algorithm 4:** Mutation.

*Input: sky*^*temp*^, *P*_*m*_, *Sky, n*
*Output: sky*^*t*^
*sky*^*t*^ *←ø*
*foreach sky*^*i*^ ∈* sky*^*temp*^
* if rand < P*_*m*_ *then*
* index = random(1, n)*
* replace randomly* }{}sky_{index,{j_{index}}}^{s{p_{index}}} *in sky*^*i*^ *by new service instance from* }{}Sky_{index}^{s{p_{index}}} *set*
* endif*
** }{}sk{y^t} \leftarrow sk{y^t} \cup sk{y^i}*endforeach*
*return* }{}sk{y^t}

The tournament method is used as an operator in the proposed genetic algorithm. The fitness function is defined as Algorithm 5, which is used to evaluate the quality of the service chain equivalent to the chromosome.

**Algorithm 5:** Fitness function.

*Input: N*_*SP*_ *, sky*^*p*^
*Output: Goal*^*p*^
sky*_i_*← ø*
*foreach sky*_*i*_^*p*^ ∈* sky*^*p*^
* Cost*^*p*^*←0 , Capacity*^*p*^*←0*
* foreach* }{}I_{i,{j_i}}^{s{p_i}} ∈* sky*_*i*_^*p*^
* Cost*^*p*^*← Cost*^*p*^*+*}{}Cos{t_{I_{i,{j_i}}^{s{p_i}}\; }} * Capacity*^*p*^ *← Capacity*^*p*^*+*}{}Capacit{y_{I_{i,{j_i}}^{s{p_i}}\; }} **
* Similarity*^*p*^*←1-|unic(*}{}I_{i,{j_i}}^{s{p_i}} *)|/N*_*SP*_
* endforeach*
* Goal*_*i*_^*p*^ *← Normal(Similarity*^*p*^*/(Cost*^*p*^*+ Capacity*^*p*^*))*
*endforeach*
*return Goal*^*p*^

### SFC context-free grammar

The CFG is discussed in this section. These CFGs can give a resilience description of the service function paths connected to provide services. For the sake of clarity, all functions that prompt services are virtualized, and then the SFs and replaceable service functions are modified. A composition with a clear sample is a single SF or an endpoint of a service flow, although it can also be complicated like a multipath structure. All CFG rules are based on mobile, data center, and security IETF use cases (Napper et al., 2018; Surendra et al., 2017; Wang et al., 2017).

The proposed CFG has four members. These members include *NN, TT, II and RR* where *NN* is a non-terminal set containing *{SFC, C, Mobile, Datacenter, Security, P, TYPE, Q, R, W, FW, LB, R, W, X, DECOMPOSITION, COMPOSITION, H, G, SF, A, B, Z}; TT* is a terminal set that includes a set of all SFs in SFC *{pgw, bng, olt, cmts, nat, dpi, mwd, part, ctrl, li, opt, tcp, opt, video, enr, head, ddos, tls, proxy, avc, ids, woc, edge, mon, adc, mon, app, seg, fw, lb, sf*_*1*_, *sf*_*2*_*,…, sf*_*n*_*}; II* is an initial symbol and equal to *{SFC};* and *RR* is a set of rules that include

1. *SFC → C SFC | C*
2. *C → MOBILE | SECURITY | DATACENTER*
3. *MOBILE → pgw P |bng P | olt P | cmts P*
4. *P → Q P|R P|W P|QR P|QW P|RW P|QRW P| λ*
5. *Q → dpi TYPE Q | LB TYPE Q |FW TYPE Q |nat TYPE Q | dpi LB TYPE Q | dpi FW TYPE Q | dpi nat TYPE Q | LB FW TYPE Q | LB nat TYPE Q | FW nat TYPE Q | dpi LB FW TYPE Q | dpi LB nat TYPE Q | dpi FW nat TYPE Q | LB FW nat TYPE Q | dpi LB FW nat TYPE Q | λ*
6. *R → li TYPE R | part:ctrl TYPE R | mwd TYPE R | li part:ctrl TYPE R | li mwd TYPE R | part:ctrl mwd TYPE R | li part:ctrl mwd TYPE R | λ*
7. *W → enr:head TYPE W | opt:video TYPE W | opt:tcp TYPE W | enr:head opt:video TYPE W| enr:head opt:tcp TYPE W | opt:video opt:tcp TYPE W | enr:head opt:video opt:tcp TYPE W | λ*
8. *SECURITY → FW-ddos TYPE | FW- tls: proxy -avc TYPE | FW-ids-ddos TYPE*
9. *DATACENTER → woc FW:edge mon adc FW:app TYPE | woc FW:edge mon FW:seg X*
10. *X → adc mon FW:app TYPE X | adc mon FW:app TYPE*
11. *TYPE→ COMPOSITION | DECOMPOSITION | SF*
12. *COMPOSITION → SF- COMPOSITION | SF-SF*
13. *DECOMPOSITION → SF: DECOMPOSITION | SF:SF*
14. *FW → fw H*
15. *H → TYPE | H TYPE*
16. *LB → lb G*
17. *G → TYPE | G TYPE*
18. *SF→ λ|sf1 A | sf2 B | sf3 C | … | sfn Z*
19. *A → λ| sf2 B | sf3 C | … | sfn Z*
20. *B → λ| sf1 A | sf3 C | … | sfn Z*
21. *C → λ| sf1 A | sf2 B | … | sfn Z*
22. *…*
23. *Z → λ| s1 A | s2 B | s3 C | … | sn-1 Y*

This CFG includes capitalized words that represent non-terminals and lowercase words that describe terminals. On the first and second lines of the CFG, you can select one of the three chains of the mobile, data center, and network security. The third, fourth, fifth, sixth, and seventh lines show how to create a mobile chain with the services. The eighth line indicates how to create a security chain with the services. The ninth and tenth lines show how to create a data center chain with the services. The eleventh, twelfth, and thirteenth lines denote how to select a service composition or decomposition. The fourteenth and fifteenth lines show how to create a firewall service. The sixteenth and seventeenth lines indicate how to create a load balancer service. In the eighteenth line up to the end, there is a structure to create a unique cycle for generating various services. All of the symbols are bng(Broadband Network Gateway), p-gw(Packet Gateway), olt(Optical Line Termination), cmts(Cable Modem Termination System), ids(Intrusion Detection System), ips(Intrusion Prevention System), e-fw(Edge Firewall), s-fw(Segment Firewall), a-fw(Application Firewall), adc(Application Delivery Controller), woc(Web Optimization Control), mon(Monitoring), fw(Firewall), lb(Load Balancing), ddos(Distributed Denial of Service), avc(Application Visibility and Control), tls(Transport Layer Security), and λ(Indicates zero occurrences of the preceding SFs).

## Evaluation

The proposed CFG-based genetic algorithm that generates the Skyline service composition (SGGA) is compared with other methods in this section. Three synthetically generated datasets were tested via the proposed method by a massive number of services and various distributions.

Each method has different behavior in different data distribution. Therefore, the performance of these methods can be evaluated in correlated, anti-correlated, and independent distributions (Morse, Patel & Jagadish, 2007). For this purpose, a usual generator was used to generate three datasets via randdataset-1.1.0:

- correlated dataset for correlated QoS
- anti-correlated dataset for anti-correlated QoS
- independent dataset for independent QoS

The following QoS-based composition methods were compared by performance:

- ILP (integer linear programming model): This is the typical global optimization method with all service competitors represented in it (Lodi & Nagarajan, 2019).
- GA: In this method, service compositions are generated by the GA with all services (Dwiardhika & Tachibana, 2019).
- KS: This signifies the K-means method used with Skyline service candidates (Kumar & Purohit, 2016).
- SGGA: This is the proposed method, which uses Skyline, CFG, and a GA, as described in the previous section.

All the measures were executed on an HP ProLiant DL380 G9 computer with 18 Intel Xeon 2.80GHz processors and 32 GB of RAM. Every single experiment was repeated 30 times, and then calculate the average value. A set of the method parameters are maximum generation 100, population size 80, crossover probability 0.85, and mutation probability 0.15. The proposed method is evaluated in the following.

### QoS assessment

**Figure 2:**
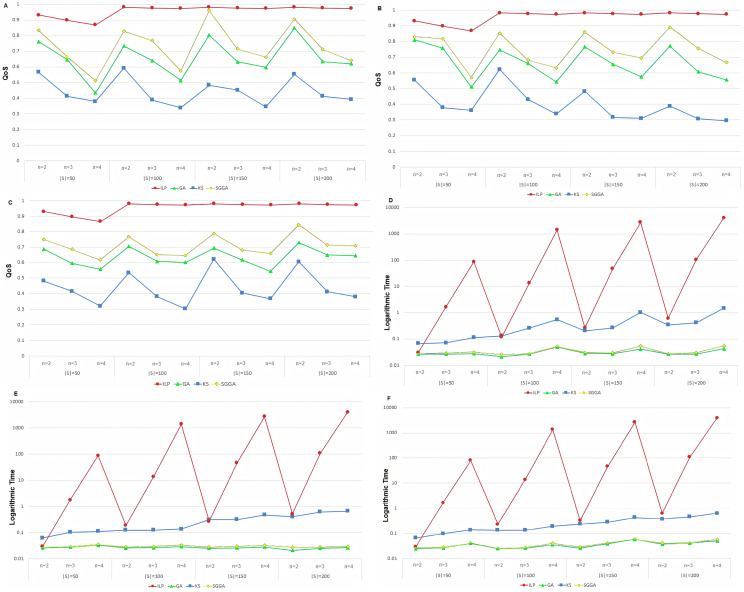
The logarithmic running time and QoS of service chain compositions in different method with anti-correlated, correlated, and independent data (A–F).

Figures 2A, 2B and 2C show the QoS of different methods with anti-correlated, correlated, and independent data, respectively. The vertical axis shows the QoS value according to Eq. (11), and the horizontal axis represents the number of services (|S|) and the length of the service chain (n).

The ILP method delivers the best quality of service because it makes all possible compositions. Therefore, this method has a higher QoS than the other three methods. The proposed method outperforms the GA and KS methods because the SGGA method follows the IETF-based standardization procedure and attempts to select the service chain with the highest QoS. Moreover, as the chain length increases, the variety of problem states increases. The proposed method reduces the service chain QoS. Increasing the chain length also reduces the number of the chains of the services matching the CFG. Simultaneously, increasing the number of available services increases the chances of creating CFG-based service compositions.

The ILP method generates all the possible cases and offers a higher QoS compared to the other methods. As the number of services, *|S|*, increases, a service chain with a higher QoS can be created due to the increased diversity of the available services. In practice, the QoS experiences a substantial decrease by decreasing and increasing the chain length. However, the increase in *|S|* can be compensated for by increasing the chain length, consequently allowing the QoS to remain unaffected.

The K-means method attempts to create a chain service with a higher QoS by classifying the services into two separated clusters and making a heap tree. In this method, similar services are placed in the cluster. The service with the highest utility service is located at the root with two clusters as its children form a heap tree recursively. As *|S|* increases, the diversity of the services increases. Therefore, services with a higher utility service are placed in the heap tree, allowing for a service chain with a higher QoS. However, the QoS of the service chain decreases as *n* increases since more services leave the heap tree and result in a reduction in their QoS.

The GA is an optimization algorithm for finding the global optimum. The search space in this algorithm is increased by increasing *n* and *|S|*. In this case, the probability of finding the global optimum at a given number of iterations decreases, making it more likely to be trapped in a local optimum. As shown in Figs. 2A, 2B and 2C, the QoS decreases by increasing *n*. As *|S|* increases, due to the increased diversity of the services, the QoS of the service chain is expected to increase, contrasting the increase in search space and the reduced probability of finding a global optimum. However, from a theoretical perspective and based on the experimental results, the GA performance improves by increasing *|S|*.

By employing grammar to reduce the search space, the proposed method finds those service chains that comply with the IETF standards compared to the GA. Although the QoS of these service chains is not necessarily higher, employing the Skyline technique and reducing the search space causes the services with a higher QoS to be used in the grammar-based GA, finally cause to increase QoS of the generated service chain.

Since the ILP method checks all possible scenarios, it has a higher execution time and has the best ones. The proposed SGGA method has a higher QoS than KS and GA. In the proposed method, the QoS using GA and the services filtering with the Skyline method was higher than the GA method. Due to the GA algorithm, the proposed method is expected to perform better than the KS method. In three methods of SGGA, KS, and GA, the QoS decreases with increasing chain length. As the chain length increases, the problem space becomes more complex, so each method's service chain is further away from the ideal chain. The proposed method, SGGA, performs better than the other two methods. As the number of services increases, the number of choices for each service in the service chain increases, which has led to the service chains with higher QoS in the GA and SGGA methods. As observed, the SGGA method's performance in various data distribution types is better than the other two methods. The behavior of the SGGA method is equivalent in different data distribution types, while the KS method depends on the data distribution.

### Running time assessment

Figures 2D, 2E and 2F illustrate the implementation of the durations of different methods with the anti-correlated, correlated, and independent data, respectively. The vertical axis shows the running time in seconds on a logarithm scale, and the horizontal axis represents the number of services (|S|) and the length of the service chain (n).

Since the ILP method assesses all the possible compositions, it has a greater runtime than the other methods and increases exponentially (refer to the order of the method) as the service chain length increases. When running the algorithm, the impact of increasing the chain length is greater than the number of services. The proposed method has a shorter execution time than the KS and ILP methods because it only examines the IETF-based compositions and reduces the problem space, reducing the algorithm runtime. According to the results, when the chain length increases, the number of services corresponding to the reduced CFG is compensated for by increasing the runtime. The runtime does not change significantly. However, when the number of available services increases, the chance of creating syntax-based service compositions increases, which is something that increases the runtime.

Since the execution time of the ILP algorithm is much longer than the other three methods, so to better show the execution time of the methods and compare them, the logarithm of the execution time in seconds has been used. The ILP method investigates all possible cases that have the time complexity of O(|S|^n^) were increasing with the increasing of |S| and n. The execution time of the other three methods increases with increasing the chain length in the number of fixed services. As the number of services increases, the execution time also increases related to the increasing problem space. It can be concluded that the number of service chains in each method depends on the chain’s length and the number of services. Also, the evaluated methods show similar behavior in different data distributions.

### Time complexity assessment

Given that *O(|S|*^*n*^*)* is the time complexity of the ILP method, increasing the *n* parameter causes the runtime to scales exponentially, as demonstrated in Figs. 2D, 2E and 2F. The time complexity of the K-means method is directly related to *|S|*, such that an increase in this parameter increases the runtime to a significant extent. Although the runtime increases by increasing *n*, it can be reduced to a shorter duration by modifying *|S|*.

Since the GA runtime is directly related to the chromosome length, *n*, an increase in *n* will increase the runtime. However, the algorithm runtime is not significantly affected by changing *|S|*. The proposed method has a longer runtime than the GA as it employs the Skyline technique and the grammar-based GA. However, compared to the GA, the proposed algorithm experiences the same trend by changing *|S|* and *n*. The time Big-O complexities of the methods are shown in Table 2. Our method’s time complexity included GA with the Skyline phase. According to Table 2, GA and SGGA have a similar time complexity and lower time complexity than the other methods.

**Table 2:** Time complexities of the methods.

Method	Time complexity
ILP	O(|S|^n^)
GA	O(maxGeneration *n*P_g_ )
KS	O(n*k*Itermax)
SGGA	O(|S|)+O(maxGeneration *n*P_g_ )

**Note:**

k is denotes the number of cluster and Itermax is equal to maximum iteration in the KS method.

### SFC context-free grammar assessment

With passing a service chain, various SFs must be used in the network, and they need to route the associating flows in a specific arrangement, resulting in different service compositions. This section presents the instances of use cases of service function chaining in mobile networks (Napper et al., 2018), data center networks (Surendra et al., 2017), and security networks (Wang et al., 2017), where the service function chain can be mixed.

**Figure 3:**
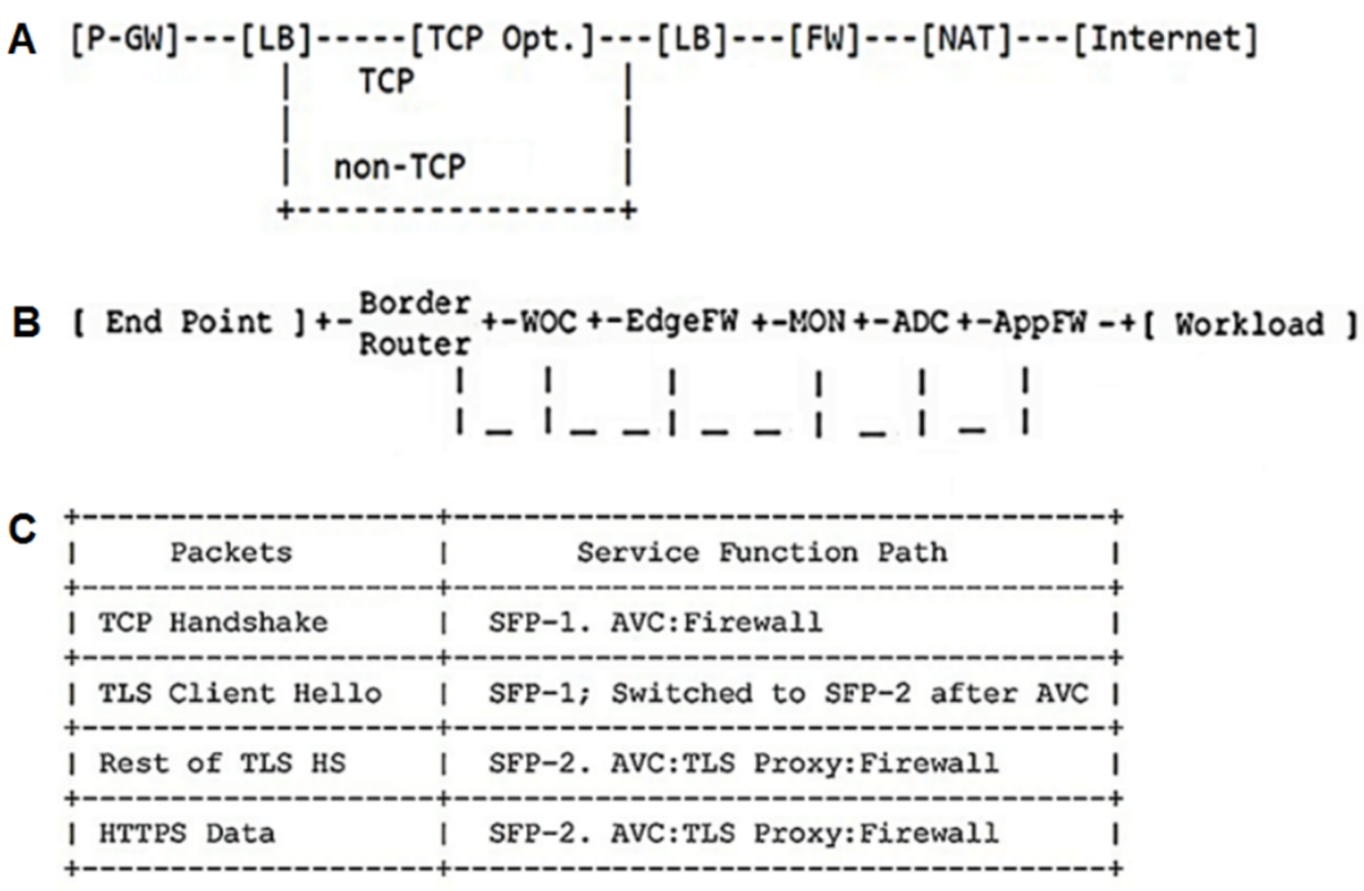
A typical service chain for mobile network (Napper et al., 2018), data center network (Surendra et al., 2017), and security network (Wang et al., 2017) defined by IETF.

The top-down traversal algorithm was employed to evaluate the proposed CFG (Reus, 2016). At each recursion level, it will visit the node first to come up with some values and pass these values to its children when calling the function recursively. So the “top-down” solution can be considered as a kind of preorder traversal (Reus, 2016). A chain was taken from the IETF mobile network to assess the proposed CFG (Fig. 3A). The result of running the TDT algorithm by the mobile chain is shown in the following expression:

*SFC → C → MOBILE → pgw P → pgw Q P → pgw LB TYPE Q P → pgw lb G TYPE Q P → pgw lb TYPE TYPE Q P → pgw lb SF TYPE Q P → pgw lb s*_*1*_ *A TYPE Q P → pgw lb tcp-opt. A TYPE Q P → pgw lb tcp-opt. λ TYPE Q P → pgw lb tcp-opt. λ SF Q P → pgw lb tcp-opt. λ λ Q P → pgw lb tcp-opt. λ λ LB TYPE Q P → pgw lb tcp-opt. λ λ lb G TYPE Q P→ pgw lb tcp-opt. λ λ lb TYPE TYPE Q P → pgw lb tcp-opt. λ λ lb SF TYPE Q P → pgw lb tcp-opt. λ λ lb λ TYPE Q P → pgw lb tcp-opt. λ λ lb λ SF Q P → pgw lb tcp-opt. λ λ lb λ λ Q P → pgw lb tcp-opt. λ λ lb λ λ FW TYPE Q P → pgw lb tcp-opt. λ λ lb λ λ fw H TYPE Q P → pgw lb tcp-opt. λ λ lb λ λ fw TYPE TYPE Q P → pgw lb tcp-opt. λ λ lb λ λ fw SF TYPE Q P → pgw lb tcp-opt. λ λ lb λ λ fw λ TYPE Q P → pgw lb tcp-opt. λ λ lb λ λ fw λ SF Q P → pgw lb tcp-opt. λ λ lb λ λ fw λ λ Q P → pgw lb tcp-opt. λ λ lb λ λ fw λ λ nat TYPE Q P → pgw lb tcp-opt. λ λ lb λ λ fw λ λ nat SF Q P → pgw lb tcp-opt.λ λ lb λ λ fw λ λ nat s*_*2*_ *B Q P → pgw lb tcp-opt. λ λ lb λ λ fw λ λ nat https B Q P → pgw lb tcp-opt. λ λ lb λ λ fw λ λ nat https λ Q P → pgw lb tcp-opt. λ λ lb λ λ fw λ λ nat https λ λ P → pgw lb tcp-opt. λ λ lb λ λ fw λ λ nat https λ λ λ*

The results indicate that the entry service chain is assumed correct and acceptable because it has reached the mobile chain from the SFC symbol. A chain was also taken from the IETF data center network to assess the proposed CFG with the data center chain (Fig. 3B). The result of running the TDT algorithm by a data center chain can be seen in the following expression:

*SFC → C → DATACENTER → woc FW:edge mon adc FW:app TYPE → woc fw H:edge mon adc FW:app TYPE → woc fw TYPE:edge mon adc FW:app TYPE → woc fw SF:edge mon adc FW:app TYPE → woc fw λ:edge mon adc FW:app TYPE → woc fw λ:edge mon adc fw H:app TYPE → woc fw λ:edge mon adc fw SF:app TYPE → woc fw λ:edge mon adc fw λ:app TYPE → woc fw λ:edge mon adc fw λ:app SF → woc fw λ:edge mon adc fw λ:app s*_*1*_ *A → woc fw λ:edge mon adc fw λ:app tcp-opt. A → woc fw λ:edge mon adc fw λ:app tcp-opt. λ*

The results indicate that the entry service chain is considered correct and acceptable because it has reached the data center chain from the SFC symbol. A chain was selected from the IETF security network to evaluate the proposed CFG with a security chain (Fig. 3C). The result of running the TDT algorithm by a security chain is presented in the following expression:

*SFC → C → SECURITY → FW- tls: proxy -avc TYPE → fw H - tls: proxy -avc TYPE → fw TYPE - tls: proxy -avc TYPE → fw SF - tls: proxy -avc TYPE → fw λ - tls: proxy -avc TYPE → fw λ - tls: proxy -avc SF → fw λ - tls: proxy -avc s*_*1*_ *A → fw λ - tls: proxy -avc tcp-opt. A → fw λ - tls: proxy -avc tcp-opt. λ*

According to the results, the entry service chain is correct and acceptable because it has reached the chain from the SFC symbol.

## Conclusions

This paper proposes a novel approach to generating the optimal composition of the service functions. The methods outlined in this paper create a service chain regardless of the standards defined in the network. The QoS-based input services were first filtered using the Skyline method to create service compositions. Then, the CFG-based genetic algorithm and the IETF generated the instance service chains. According to the presented context, the results showed that the offered CFG could significantly decrease the runtime and increase the QoS of service compositions compared to other methods. The experimental results showed that the proposed method outperforms previous methods concerning the service quality, running time, and time complexity. Ultimately, the proposed CFG can be generalized to other SFC use cases.

In the proposed method, due to the use of grammar, compared with other methods, the number of service compositions is reduced so that the execution speed of the algorithm increases. The obtained service compositions are also valid. In the case of QoS, however, there is no guarantee that the proposed method will work better, as there may be invalid service compositions with high QoS, but these have been removed by grammar.

## Future works

Other formal language types, such as the Turing machine, can filter out invalid chains for future work. Also, different IETF types use cases can be used to select the appropriate chains. The proposed method can also be developed for the social network using communities.

## Supplemental Information

10.7717/peerj-cs.603/supp-1 Supplemental Information 1 Code.

10.7717/peerj-cs.603/supp-2 Supplemental Information 2 Data.

10.7717/peerj-cs.603/supp-3 Supplemental Information 3 Execution file.

10.7717/peerj-cs.603/supp-4 Supplemental Information 4 Grammar.

## Additional Information and Declarations

### Data Availability

The following information was supplied regarding data availability: Raw data and code are available as Supplemental Files.
